# Evaluation of Various Metallic Coatings on Steel to Mitigate Biofilm Formation

**DOI:** 10.3390/ijms10020559

**Published:** 2009-02-12

**Authors:** Hideyuki Kanematsu, Hajime Ikigai, Michiko Yoshitake

**Affiliations:** 1 Dept. MS & E, Suzuka National College of Technology (SNCT) / Shiroko-cho, Suzuka Mie, 510-0294, Japan; 2 Dept. Chem & Biochem, Suzuka National College of Technology (SNCT)/ Shiroko-cho, Suzuka Mie, 510-0294, Japan; E-Mail: ikigai@chem.suzuka-ct.ac.jp; 3 Advanced Electronic Materials Center, National Institute for Materials Science (NIMS)/ 3-13, Sakura, Tsukuba, Ibaraki 305-0003, Japan; E-Mail: yoshitake.michiko@nims.go.jp

**Keywords:** Biofilm, alloy plating, HSSL process, tin-silver alloy, Pseudomonas aeruginosa, Pseudoalteromonas carageenovara, biofouling

## Abstract

In marine environments and water systems, it is easy for many structures to form biofilms on their surfaces and to be deteriorated due to the corrosion caused by biofilm formation by bacteria. The authors have investigated the antibacterial effects of metallic elements in practical steels so far to solve food-related problems, using *Escherichia coli* and *Staphylococcus aureus*. However, from the viewpoint of material deterioration caused by bacteria and their antifouling measures, we should consider the biofilm behavior as aggregate rather than individual bacterium. Therefore, we picked up *Pseudomonas aeruginosa* and *Pseudoalteromonas carageenovara* in this study, since they easily form biofilms in estuarine and marine environments. We investigated what kind of metallic elements could inhibit the biofilm formation at first and then discussed how the thin films of those inhibitory elements on steels could affect biofilm formation. The information would lead to the establishment of effective antifouling measures against corrosion in estuarine and marine environments.

## 1. Introduction

The importance of biofilm formation and its inhibition and mitigation technology have been recognized from the industrial viewpoint only gradually, since people have recognized the important role of biofilm to deteriorate the function of materials and their structures [1,2]. The corrosion of materials caused by biofilm formation and the following growth would lead to maintenance and repair of marine structures, water systems etc, and the costs can sometimes be huge. Therefore, it is very important, not only from a scientific viewpoint, but also from an economical one, to clarify the mechanisms of biofilm formation, and also to establish techniques for their inhibition and mitigation.

A biofilm is an assemblage of microorganisms that is irreversibly associated with a material surface and enclosed in a matrix composed primarily of polysaccharides [3]. Usually, bacteria tend to gather together and form a micro-space at the surface of various materials surrounded by the slime they secrete, as biofilm can make them resist more easily against some “foreign enemy” such as biocides, or fluid movements which could kill them or forcibly remove them. They would also get a chance to have access to nutrients in the biofilm more easily, even in some oligotrophic environments. In fact, biofilm can be a strategic measure for bacteria to effectively survive in any environment [4].

Since the biofilm is formed on the material surface, the interaction between materials and bacteria should be very important. Characklis *et al.* [5–8] proposed that a very thin organic film, which he called a “conditioning layer”, is formed on material surfaces and would attract bacteria in the beginning stage. According to his model, pioneer bacteria which are highly motile due to their flagella would move toward the material surface driven by the concentration gradient of organic matter (chemotaxis) [9]. The pioneer bacteria would repeat the physical adsorption and desorption process many times and finally they would stick to the material surfaces by sticky extracellular polymeric substances (EPS or Glycocalyx) they would secrete [10]. Then other bacteria (secondary colonizers) would join the pioneer ones to form the biofilm [11]. Even though some researchers concluded that the material surface has little or no effect on biofilm development [12], all of these mechanism mentioned above suggest strongly that the formation stage should be influenced by mutual interactions between material surfaces and bacteria [13]. In other words, the inhibition of biofilm formation at the beginning stage could lead to an effective mitigation technique against the subsequent slime formation, microbial corrosion and biofouling. In fact, the authors have found in some pilot studies so far that some metallic elements could inhibit biofilm formation [14]. In the study, tin-silver alloy plated steel was not stained with crystal violet, after it had been immersed in a *Pseudomonas aeruginosa* culture fluid for a certain time, suggesting that the there was no formation of biofilms on the alloy film. Since the study was not systematic for biofilm formation, detailed information and knowledge were needed to investigate and confirm the inhibition effect of metallic elements against biofilm formation on the basis of tin-silver alloy plating and some other ones.

Therefore, the authors have investigated and now clarify in this paper what kind of metallic elements could inhibit biofilm formation with different surface finished specimens at first and then discuss how the thin films of those inhibitory elements on steel could affect biofilm formation. As bacteria, we chose *Pseudomonas aeruginosa*, since they have high capability to form biofilms, which in some cases can begin in as little as 30 seconds [15]. Since they are very motile due to their flagella and they exist in most environments, they can be the pioneer bacteria involved at the beginning stage of biofilm formations in many environments. In addition to *P. aeruginosa*, *Ps. carageenovara* was selected for the current study, since they can also easily form biofilms in marine environments. As for the use of the latter bacteria, we were deeply aware of the possible application of the results to problems in marine structures in the future. Even though the data and knowledge obtained in the current study were very fundamental at this point, they are expected to be highly applicable to practical inhibition or mitigation techniques against the biofilm formation and development for water systems and marine structures.

## 2. Results and Discussion

### 2.1. Material surface phases

First of all, the surface phases for specimens used in the current study were investigated and confirmed. It is very important to identify the surface phases and to clarify their structures, since biofilms are very closely associated with material surfaces.

Specimen b is an iron based carbon steel. Therefore, iron is absolutely the primary element existing on the surface layer which interacts with environmental factors, including bacteria. By the same token, the pure metal film specimens c, d, h, i and j have their pure single elements as surface phases, respectively. All of this was confirmed by X-ray diffraction (XRD) analysis. From the electrochemical viewpoint, the dissolution characteristics for plated specimens are different from that for the substrate specimen.

**Figure 1:**
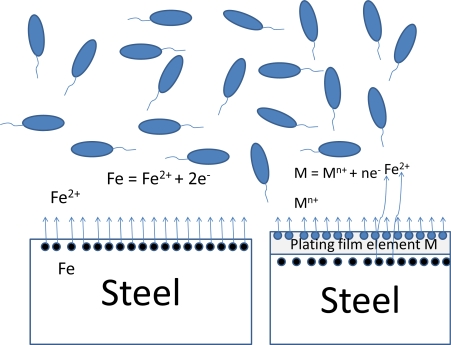
Schematic illustration of surface reactions at the interface between metals and environments.

Figure 1 shows schematically the equilibrium reactions between the surface phase and the moist environment in the case of substrate and pure metal film. In the case of substrate specimen, iron dissolves into environment to some extent as iron ion and it interacts with bacteria. In the case of a plating film specimen, the primary reaction should be the ionic dissociation of the plating film element, M. However, the substrate element, iron, might be ionized and dissolved into the environment to some extent, since the surface phase would have some microscopic crevices or pores and the substrate iron could have a contact with the environmental liquid through the micro-defects. The extent depends on the cases. On the other hand, the specimens having plural surface phases would behave differently.

**Figure 2:**
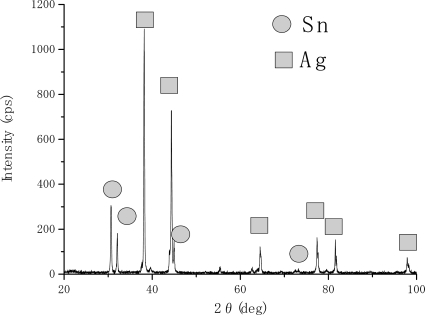
XRD result for specimen e.

**Figure 3:**
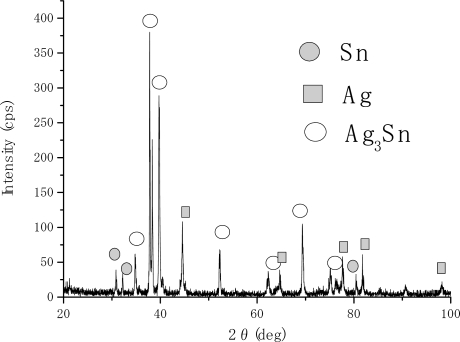
XRD result for specimen f.

Figures 2 and 3 show the XRD analyses results for stacked single layer specimens of tin and silver, respectively. The vertical axis corresponds to the X-ray intensity (cps: counts per second) and the horizontal one to the diffraction angle. Figure 2 shows the result for specimen e (see Table 2). All of the peaks observed in this figure correspond to tin and silver peaks. The results suggest that the stacked single layers on the steel substrate formed and any reactions between the two layers did not occur. Figure 3 shows the XRD result for specimen f (see Table 2). In this figure, tin peaks were still observed and it suggests that some tin parts remained, without forming compounds.

On the other hand, silver peaks and tin-silver intermetallic compound peaks were observed in this case. When the specimen was heat treated, mutual diffusion between the surface single phases occurred and intermetallic compound phases were formed. The XRD result shown in Figure 3 suggests that the surface layers were composed of unreacted tin, silver and the intermetallic compound phase (Ag_3_Sn). And it suggests also that those phases formed layer by layer in the order of tin, intermetallic compound and silver phase from the surface to the inner of specimens.

**Figure 4:**
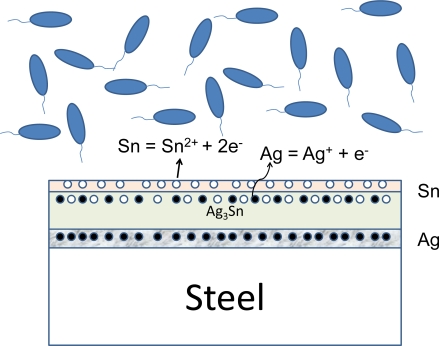
Schematic illustration of the surface structure for Sn-Ag alloy film produced by heat treatment.

Figure 4 shows the constitution of the surface phases and their electrochemical behaviors with the environment summarized schematically. Even though a pure tin phase and a silver one still remained in the surface films, the intermetallic compound phase was dominant. In addition to tin ion, it would appear that silver ion from the second phase was dissolved into the environment. As for silver ion, the silver element comes from the intermetallic compound, Ag_3_Sn. Therefore, the dissociation of the compound should be considered at first, strictly speaking. However, all of these detailed processes are still unknown at this point and the mechanism as ionization at material surface and the following interactions between ions and bacteria are hypothesized in this paper.

### 2.2. Antibacterial effects of some metals

In our previous study, antibacterial effects of some metal elements against bacteria were investigated. One of the tests for antibacterial effects used metal powders. The change of colony numbers with time was measured, when those powders were immersed with bacteria such as *Escherichia coli*, *Klebsiella pneumoniae* and *Staphylococcus aureus* [16, 17]. According to the results, cobalt, copper and zinc showed very high antibacterial effects again those bacteria. Silver showed also relatively high antibacterial effect, however the extent was smaller than that of cobalt, copper and zinc. Iron did not show any significant antibacterial effect at all. It would appear that the antibacterial effect would be a result of the direct contact between bacterial cells and ionized ions dissociated from the metal powders in culture media solutions. The results suggest that these heavy metal ions such as silver, zinc, copper and cobalt showing antibacterial effects could bring about enzyme inhibition in the outer membranes of bacteria cells.

Even though the results of this Metal Powder Screening Test could provide fundamental data to screen antibacterial metal elements from non-antibacterial ones, one should seek another testing method to evaluate the antibacterial effect of items that approximate commercial products more correctly (ISO22196). The test is usually called the Film Covering Method and it provides us the useful information needed for practical applications. The specimens were put in a plastic Petri dish, while the bacterial solution was prepared as follows: the bacteria were incubated in 10 mL of a nutrient broth for 24 hours at 35 °C, and then diluted two-thousand fold with sterilized water and established as a bacterial solution. The diluted bacterial solution was applied to the specimen (16 μL/cm) and then a polymer film was laid over the solution. The sample was kept in an incubator for 24 hours at 35 °C. After the incubation, a solution of 10 mL of sterilized water containing 200 μL of Tween® 80 (a nonionic surfactant and emulsifier) was introduced into the plastic Petri dish and the bacteria attached to the specimen and polyethylene film were washed into the aqueous solution. To determine the number of viable cells, serial decimal dilutions of the cell suspension were made, a 0.1 mL portion of which was uniformly spread on an agar medium. The plate was incubated at 35 °C for 24 hours and the colonies formed were counted. The viable cell count was represented as colony forming unit per milliliter (CFU/mL). The final colony formation number was measured to evaluate the antibacterial properties. Table 1 shows the result of Film Covering Method for tin plated specimen (specimen c). The bacteria used for the test was *E. coli* ATCC 25922. Even after a certain time passed, the numbers of colony did not decrease significantly and it indicates clearly that tin did not show any antibacterial effects against *E. coli*.

However, alloying of tin film with some antibacterial elements can increase the antibacterial effect significantly. In fact, the tin-copper alloy film steel [18, 19] and the tin-silver alloy film steel [19–21] made by the Heating Stacked Single Layer Process (HSSL Process) [22, 23] showed remarkable antibacterial effects. In both cases, intermetallic compounds of tin and copper or silver were produced on the surfaces of steels. As shown schematically in Figure 4, it would appear that the antibacterial elements dissociated from the intermetallic compound phases would be ionized to inhibit the enzyme activities of bacterial cells. And as a result, the antibacterial effect was developed.

### 2.3. Inhibition capability of biofilm formation

The results obtained in the previous section (Film Covering Method and Metal Powder Screening Test) only showed the antibacterial characteristics of metallic elements or products against single species of bacteria and it only provides information about the antibacterial effects against planktonic bacteria, even though it is also very effective and fundamental information for practical applications.

However, most of bacteria in water systems, for example, exist in biofilms and planktonic bacteria constitute only a small portion of them [24], and since it is quite natural to deal with the interaction among plural, complicated groups of bacteria and materials for practical application, the evaluation of inhibition capabilities against biofilm formations is essential for practical applications for the mitigation or inhibition techniques against the biofilm formation.

**Figure 5:**
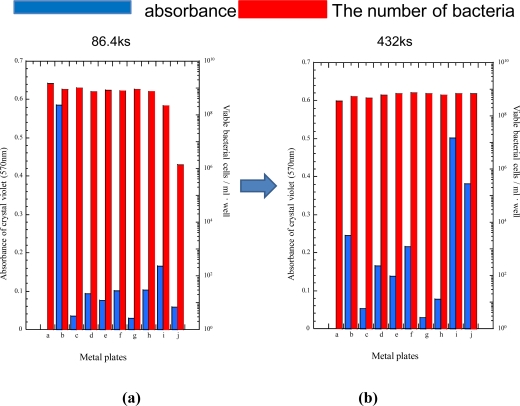
Biofilm formation of *Pseudomonas aeruginosa* on surface of various specimens. (a) immersion time: 24 hours; (b) immersion time: 120 hours.

Figure 5 shows the absorbance of crystal violet staining biofilm on specimens and the number of viable bacterial cells in the case of *P. aeruginosa*. The number of viable cells corresponds to the antibacterial effects, when the dissolved ions co-existed with the bacteria in wells. As for the antibacterial effects (the number of viable bacteria cells), there were no significant differences among most specimens, except for the cobalt plated specimen at 24 hours. However, the number of viable bacterial cells in the case of both specimens also increased to the same level for other specimens. On the other hand, the inhibition capability against the biofilm formation corresponding to the absorbance of crystal violet changed from specimen to specimen drastically. In general, the absorbance of crystal violet is large when the biofilm formation is also large in extent. Therefore, the absorbance can be defined as the inhibition capability against the biofilm formation. From the viewpoint, Figure 5 shows clearly that iron substrate could grow biofilm within 24 hours very easily (specimen b). All of other specimens increased the inhibition capabilities, being compared with iron specimen. Particularly, the tin plated specimen (specimen c) and stainless steel specimen (specimen g) showed very high inhibition capabilities and the effects continued through the immersion time of 120 hours, even though the absorbance of crystal violet for most of specimens increased to some extent. For zinc (specimen i) and cobalt plated specimens (specimen j), the inhibition capability decreased with the increase of immersion time. For the cobalt plated specimens, the decrease could be attributed to that of antibacterial effect as shown in Figure 5. For the zinc plated specimens, chemical reactions between the zinc layer and culture solution might change the surface properties (the conditioning layer) for the attachment of bacteria. In fact, a sort of precipitation was observed in the culture medium, even though it has not been identified yet. The silver-tin alloy film specimen (specimen f) showed relatively high inhibition capability against biofilm formation. However, the cause might be attributed not to the well known antibacterial effects of silver, but rather than to the tin having very high inhibition capability against biofilm formation. Specimen e had the tin layer on the top of surface layers as well as the pure tin plated specimen (specimen c). However, the inhibition capability of biofilm formation for the former was smaller than that for the latter. These tendencies suggest that not only antibacterial effects, but also different material factors would affect the inhibition capabilities against the biofilm formation. The assumption should be experimentally investigated further from various viewpoints, which would be an important topic in the future.

**Figure 6:**
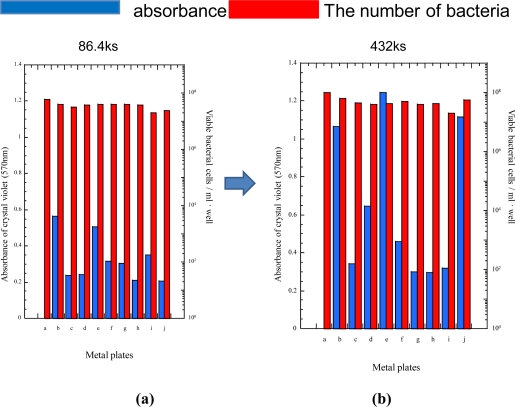
Biofilm formation of *Pseudoalteromonas carageenovara* on surfaces of various specimens. (a) immersion time: 24 hours; (b) immersion time: 120 hours.

The results of similar measurements for *Ps. carageenovara* are shown in Figure 6. As for the antibacterial effects corresponding to the number of viable bacteria cells, the tendency was very similar to that for *P. aeruginosa*. Also in this case, the inhibition capabilities against biofilm formation did not correlate directly with antibacterial effects. The tin plated specimen (specimen c) showed high inhibition capability against biofilm formation as well as the silver (specimen d), copper (specimen h), zinc (specimen i), cobalt (specimen j) plated specimens. However, the tin plated specimen having the silver underlayer (specimen e) showed lower biofilm formation inhibition capability, and the inhibition capability decreased drastically with the increase of immersion time [Figure 6-(b)]. Stainless steel showed high inhibition capability also in the case of *Ps. carageenovara* and the capability continued with the increase of immersion time. From the viewpoint of enduring the inhibition capability, tin plated specimen having the underlayer of silver (specimen e) and cobalt plated specimen (specimen j) were the worst.

Since the antibacterial effect did not have good correlations with the inhibition capability against biofilm formation in both cases, it would appear that the cause of the inhibition at the initial stage of biofilm formation should be attributed to some other material factors. Unfortunately, the cause could not be determined in this study. However, some guidelines for the future potential studies in the area can be mentioned at this point, considering the results and tendencies mentioned above.

Fouling processes in various environments would follow the biofilm formation. In marine environments and some water systems, people often say that the biofilm would be inevitably formed on materials, whatever countermeasure technique would be used. When the statement is related to the growth stage of biofilm or to a complete inhibition technique, they may be correct. However, the formation of biofilm can be delayed by some appropriate surface modification or treatment for metallic materials including steels which would lead to some effective mitigation of biofilm formation and development. For example, tin showed high inhibition capability against biofilm formation in our study. Probably, the plating could change the surface charge which would affect the adsorption-desorption of pioneer bacteria, or it might constrain the surface conditioning stage by the adsorption of organic carbon onto material surfaces negatively. Conventionally, the surface smoothness has been mentioned as an important growth constraint for biofilm formation [25]. However, most of specimens except for alloy specimens had almost same surface smoothness in this study and the immersion of specimens in the fluidized bacterial solution was not dynamic, but rather quite static. Therefore, those factors which could constrain the attachment of pioneer bacteria otherwise did not work effectively.

We certainly have to find some appropriate antibacterial elements for the purpose of inhibition against biofilm formation in the future. However, it is much more important for us to find some nontoxic elements in the environment, such as tin, which can be used in combination with different elements as alloy film. They can delay the biofilm formation and mitigate the development, which would lead the constraint of fouling on many structures in marine environments and water systems as a result.

## 3. Experimental

### 3.1. Specimens

Specimens used in this study are summarized in Table 2. As bulk materials, carbon steels (JIS SS400: specimen b, C: 0.18 wt%, Mn: 0.5 wt%, Si: 0.3 wt%, P: 0.04 wt%, S: 0.04 wt%, Cu: 0.035 wt%) and stainless steel (JIS SUS304: specimen g) were used. On the other hand, some metal and alloy films were formed on steels (JIS SS400) in different ways. As pure metal electroplating film, tin plating from a sulfate bath (specimen c), silver plating from a cyanide bath (specimen d), copper plating from a sulfate bath (specimen h), zinc plating also from a cyanide bath (specimen i) and cobalt plating from a chloride bath were chosen. All of them were prepared by outside suppliers for their commercial uses.

As alloy films, tin-silver plated specimens (substrates were also JIS SS400) were prepared through the HSSL (Heating Stacked Single Layers) process. The detailed procedures for the process in this study were carried out as follows: the silver film was electrolytic-plated on the steel from a cyanide solution. Also in this case, they were prepared by an outside supplier. Then tin was formed on the silver plated steels by the following sputtering process: a tin plate (152×76 mm, 3 mm thickness) was prepared as the sputtering target. Using the target, radio frequency sputtering of tin was carried out in a 0.67 Pa argon atmosphere. The output power was kept at 200 W during the sputtering process. The sputtering time was changed so that thin tin films thickness was about 0.5 micrometers. Then the stacked single layers specimens of tin and silver on steels were heat treated in an electric muffle furnace (Muffle type furnace FP31, Yamato Co.). The heat treatment temperature was 200 °C and the heat treatment time was 3 hours. After the heat treatment, specimens were cooled in air. The atmosphere of the furnace was not regulated specifically and all of these heat treatments were carried out under the atmospheric pressure.

### 3.2. XRD measurement

The surface structures for all specimens were analyzed by X-ray diffraction analysis (XRD). The apparatus used was a Rigaku Co. RINT 2100 diffractometer. The electrode was copper. The X-ray voltage was 40 kV and the current 20 mA. The peaks were measured in the range from 20° to 100°. The scan rate for the diffraction was 2°/min. The diffraction data were analyzed with a commercial software package (Match! Ver.1.9, Crystal Impact Co.).

### 3.3. Evaluation for biofilm formation and antibacterial effect

The bacteria used were *P. aeruginosa* PA01 and *Ps. carageenovara* NBRC12985. The former has been well known for the high capability for formation of biofilms and exists in ambient environments universally. The latter also has high biofilm formation capability and its niche is mainly the marine environment. *P. aeruginosa* and *Ps. carageenovara* were cultivated in 5 mL of nutrient broth at 35°C for 24 hours and PYMS broth (polypeptone, 10 g; yeast extract, 2 g; MgSO_4_·7H_2_O, 0.5 g; artificial seawater, 36 g; ultra pure water, 1,000 mL; pH 7.2) at 25 °C for 24 hours, respectively. Each bacterial cell adjusted approximately to 10^3^ cells/mL was suspended in 2-fold diluted nutrient broth and PYMS one, respectively. Two mL of bacterial suspension were poured and metal plates disinfected by 75% ethyl alcohol were placed at a slanted position into each of a 24 well flat bottom plate. Two sets of them were prepared and both kinds of bacteria were grown statically at 35 °C and 25 °C for the predetermined time, respectively. In a preparatory experiment, the biofilm formed clearly on carbon steels at 24 hours and did not change remarkably till 72 hours. Therefore, 24 hours was when the biofilm formation was expected, and 72 hours, well beyond the maximum time in the preparatory experiment, were selected as cultivation times to observe any drastic changes. After the predetermined cultivation time passed, they were taken out of an incubator and then bacterial cell numbers in culture were measured, using assay for colony forming unit on agar. At the same time, the biofilm formation on a surface of each metal plate was observed by crystal violet staining. The crystal violet staining assay was carried out as follows: the metal plates taken out of the previous wells were placed in new ones filled with ultrapure water. After 5 minutes the metal plates were rinsed with ultrapure water and 1 mL of 0.2% crystal violet solution was added to each well. The biofilm was stained in 1 hour at room temperature, and the plates taken out of the wells finally were rinsed five times with ultrapure water. Crystal violet attached to the back and lateral sides of metal plates were removed by cotton buds. The crystal violet on the surface of metal plates was dissolved in 95% ethyl alcohol and absorbance of the dissolved solution (100 μL) poured in well of a 96 well flat bottom plate was measured at the wavelength of 570 nm using a microplate reader (BIO-RAD, Model 550). Antibacterial effect of each metal element was performed with evaluation of Film Covering Method (ISO22196) [16].

## 4. Conclusions

The antibacterial effects and the inhibition capability against biofilm formation of some plated steels were investigated. The bacteria used as targets in this study were *P. aeruginosa* and *Ps. carageenovara.* Nine kinds of steel specimens were immersed into the bacteria solutions. After the predetermined time passed, the antibacterial effect was evaluated by the number of viable bacterial cells in the solution. The inhibition capability against the biofilm formation was assessed by measuring the absorbance of crystal violet staining the specimens. The following results were obtained in this study.

1. At the beginning of biofilm formation (within 24 hours), plated steel by tin, silver, copper, zinc and cobalt showed high inhibition capability against the formation of biofilms. Stainless steels also showed high inhibition capability. Even though the alloy film of tin and silver also shows higher inhibition capability, the extent for the alloy film became smaller than that of these single elements.
2. The inhibition capability against biofilm formation decreased with the increase of immersion time to some extent. The extent differed from specimen to specimen.
3. The antibacterial effects were not recognized remarkably for all specimens, except for cobalt and zinc plated specimens within 24 hours. The antibacterial effects did not have any direct correlation with the inhibition capability against the biofilm formation.
4. It would appear that the inhibition capability against the biofilm formation depended on the material factors affecting the attachment of bacteria onto them at the beginning stage.

## Figures and Tables

**Table 1:** Antibacterial effects for tin plated steel measured by Film Covering Method.

time (s)	specimen	*E. coli* ATCC25922
0	control	9.50 × 10^4^/polystyrene plate
86.4×10^3^	control	2.04 × 10^6^/polystyrene plate
	tin plated steel (specimen c)	7.51 × 10^5^/metal plate

**Table 2:** Specimens used in this study.

symbol	contents
a	control (without spec.)
b	carbon steel (JIS SS400)
c	tin plated steel (film thickness 10 micrometer)
d	silver plated steel
e	tin-silver alloy specimen (without heat treatment)
f	tin-silver alloy specimen heat treated in 473 K for 10.8 ks
g	stainless steel (JIS SUS304)
h	copper plated specimen
i	zinc plated specimen
j	cobalt plated specimen
